# Event Related Potential Study of Language Interaction in Bilingual Aphasia Patients

**DOI:** 10.3389/fnhum.2018.00081

**Published:** 2018-03-05

**Authors:** Elvira Khachatryan, Benjamin Wittevrongel, Kim De Keyser, Miet De Letter, Marc M. Van Hulle

**Affiliations:** ^1^Laboratory for Neuro- and Psychophysiology, Department of Neuroscience, KU Leuven, Leuven, Belgium; ^2^Department of Speech, Language and Hearing Sciences, Ghent University, Ghent, Belgium

**Keywords:** bilingual aphasia, event-related potentials, language interaction, language exposure, language proficiency

## Abstract

Half of the global population can be considered bilingual. Nevertheless when faced with patients with aphasia, clinicians and therapists usually ignore the patient’s second language (L2) albeit its interference in first language (L1) processing has been shown. The excellent temporal resolution by which each individual linguistic component can be gaged during word-processing, promoted the event-related potential (ERP) technique for studying language processing in healthy bilinguals and monolingual aphasia patients. However, this technique has not yet been applied in the context of bilingual aphasia. In the current study, we report on L2 interference in L1 processing using the ERP technique in bilingual aphasia. We tested four bilingual- and one trilingual patients with aphasia, as well as several young and older (age-matched with patients) healthy subjects as controls. We recorded ERPs when subjects were engaged in a semantic association judgment task on 122 related and 122 unrelated Dutch word-pairs (prime and target words). In 61 related and 61 unrelated word-pairs, an inter-lingual homograph was used as prime. In these word-pairs, when the target was unrelated to the prime in Dutch (L1), it was associated to the English (L2) meaning of the homograph. Results showed a significant effect of homograph use as a prime on early and/or late ERPs in response to word-pairs related in Dutch or English. Each patient presented a unique pattern of L2 interference in L1 processing as reflected by his/her ERP image. These interferences depended on the patient’s pre- and post-morbid L2 proficiency. When the proficiency was high, the L2 interference in L1 processing was higher. Furthermore, the mechanism of interference in patients that were pre-morbidly highly proficient in L2 additionally depended on the frequency of pre-morbid L2 exposure. In summary, we showed that the mechanism behind L2 interference in L1 processing in bilingual patients with aphasia depends on a complex interaction between pre- and post-morbid L2 proficiency, pre- and post-morbid L2 exposure, impairment and the presented stimulus (inter-lingual homographs). Our ERP study complements the usually adopted behavioral approach by providing new insights into language interactions on the level of individual linguistic components in bilingual patients with aphasia.

## Introduction

The incidence rate of bilingual aphasia is keeping pace with the increase in bilingualism which is currently exceeding 50% of the global population (Grosjean, 1994). However, the pathological and compensatory mechanisms behind it have been sparsely studied. When encountering patients with aphasia, clinicians and therapists usually ignore the patient’s second language (L2) and concentrate on recovering the first language (L1), especially when it is the dominant one.

The interaction between two languages in the bilingual brain is still a controversial topic and the question whether two languages are activated simultaneously when processing only one language (Rodriguez-Fornells et al., 2002; Carrasco-Ortiz et al., 2012) still needs to be resolved. The interference of L2 in L1 processing was studied repetitively for healthy bilinguals using both electrophysiological (EEG, MEG) and behavioral methods (Caramazza and Brones, 1979; Midgley et al., 2008; Martin et al., 2009). For bilingual patients with aphasia this issue was studied using behavioral methods only (Kiran and Iakupova, 2011; Siyambalapitiya et al., 2013; Kiran et al., 2014). According to these studies, L2 interference and language interaction in general depends on a complex interaction between pre- and post-morbid proficiency levels, impairment location, severity level and type of developed aphasia. Recently, Verreyt et al. (2013) showed that bilingual individuals with differential aphasia (one language is more impaired than the other) suffer from impairment of cognitive control. Here, the more impaired language interfered with the processing of the preserved one only when cross-language competition demands were low. So far, the patients included in those studies were pre-morbidly balanced bilinguals, as they were living and working in an either L2 or bilingual environment pre- and post-morbidly. Since a considerable number of bilinguals can be referred to as dominant (prefer one language over the other) (Baker and Prys Jones, 1998; Birdsong, 2014), studying dominant bilinguals with aphasia seems a logical and important goal. We will report here on results obtained from patients who were less proficient in their L2 both before and after the development of aphasia (L1 dominant) and acquired their L2 when they were around 10 – 12 years old (late bilinguals).

Previous studies with bilingual patients with aphasia investigated language interference mainly by using translation paradigms or inter-lingual cognates (words with the same semantic and lexical representations across languages; e.g., word “piano” has the same meaning in English, French and Dutch) (Roberts and Deslauriers, 1999; Siyambalapitiya et al., 2013). Even though these paradigms can serve as a good starting point for studying language interference, it was recently argued (Wu and Thierry, 2010) that translation paradigms force both languages to be activated at the same level, rendering the interaction unavoidable. On the other hand, inter-lingual cognates do not activate both languages simultaneously but they were suggested (Cristoffanini et al., 1986; Davis et al., 2010; Comesaña et al., 2015) to belong to a special group of words outside both lexicons and, hence, could be processed differently from “normal” words of each lexicon. In this study, we employ inter-lingual homographs in order to evaluate the interference of L2 in L1 processing. Despite their lexical overlap, inter-lingual homographs do not overlap in the semantic domain (e.g., the word “angel” means “sting” in Dutch), thus they can be processed as separate lexical entities in each lexicon. Therefore, the evaluation of the experimental paradigm involving them will give us a clearer overview on L2 interference in L1 processing in bilingual patients with aphasia. The studies on healthy bilinguals showed that depending on experimental conditions, such as task (Dijkstra et al., 1998), type of stimulus (van Heuven et al., 1998) and even one’s awareness about the importance of L2 in the experiment (Khachatryan et al., 2016), the processing of inter-lingual homograph can be facilitated, inhibited or processed similar to any other word of the studied lexicon.

Unlike previous bilingual aphasia studies, which were solely relying on behavioral data (Kiran and Iakupova, 2011; Gray and Kiran, 2013; Siyambalapitiya et al., 2013), we will consider in addition to behavioral results of task performance, the electrophysiological signature in response to our stimuli using the event-related potential (ERP) technique. As language processing (comprehension) in the brain unfolds extremely fast (i.e., a matter of milliseconds), discriminating its individual components (semantics, syntax, morphology) requires a recording technique with at least similar temporal resolution. The behavioral response is a more indirect way to evaluate language processing, as it takes place long after the processing of individual linguistic components (e.g., lexical access, semantic processing). Furthermore, in behavioral studies on language processing, the collected data could be confounded by a number of factors, especially in the case of patients (general slowing, confusion, difficulty of the task). ERP is a technique with excellent temporal resolution that can monitor brain activity with millisecond precision. They are transient positive or negative deflections in EEG amplitude, time-locked to an external stimulus (Luck, 2005). Although this technique has been used for more than three decades to study language processing in both healthy (mono- and bilingual) individuals and patients with aphasia (Kutas and Federmeier, 2011), we are not aware of any successful ERP study on language interaction in bilingual patients with aphasia.

The ERPs reflecting different components of language processing (orthography, phonology, lexical access, semantic processing, etc.) were previously studied in the context of both healthy (mono- and bilinguals) (for review, see Friederici, 2002; Moreno et al., 2008) and aphasia (monolingual) (Hagoort et al., 1996; Kaan, 2007). The most frequently used ERP components in studies on word processing (a single word or word pair paradigm in both healthy subjects and aphasia patients) are the P200 and N400. The P200 is a positive going potential peaking around 200 ms after the onset of the stimulus and reflecting the processing of orthography and phonology (Comesaña et al., 2012), conflict activation during processing of mentioned modes (pre-lexical stage) (Landi et al., 2007), and, as more recently suggested, lexical access (Coulson et al., 2005; Lee et al., 2012). The N400 (Kutas and Federmeier, 2009), on the other hand, is a negative going potential that reflects the processing of a potentially meaningful stimulus in general and in particular, semantics in linguistics. Its amplitude increases in response to semantic violation in a sentence context or a word unrelated to the previously presented one in a word-pair paradigm (Kutas and Federmeier, 2011). For L2, the N400 amplitude also increases in response to semantic violation, however, in this case, the amplitude is in general smaller compared to L1 and the peak latency is longer (Weber-fox and Neville, 1996; Hahne, 2001).

For patients suffering from aphasia, depending on the severity level, the amplitudes and latencies of these ERPs differ from those of healthy subjects. For instance, in patients with acute aphasia, when performing a lexical decision task, their P200 was larger and more delayed compared to healthy controls (Aerts et al., 2015). Patients in a chronic stage of aphasia with a severe comprehension deficit also exhibit a larger P200 amplitude in response to semantically violated sentences compared to congruent sentences, unlike the ones with mild comprehension deficit (Kawohl et al., 2010) where no difference in P200 amplitudes was observed. As to the N400, patients with spared comprehension exhibit an N400 similar to healthy controls (Hagoort et al., 1996), or only slightly delayed in latency (Khachatryan et al., 2017), while those with impaired comprehension (moderate to severe) might exhibit an N400 with decreased amplitude and delayed latency (Hagoort et al., 1996; Swaab et al., 1997) or no N400 at all (Kawohl et al., 2010). Since the ERPs of patients with mild comprehension deficit were shown to be similar in terms of amplitudes and latencies to those of healthy individuals (Hagoort et al., 1996), and since the patients we tested, also had mild comprehension deficit (see further in section “Subjects”), we expect this similarity in ERPs to also pertain to our study. However, we expect these ERPs to differ when we compare responses to different stimulus groups.

The N400 is also the main ERP studied in healthy bilinguals during inter-lingual homograph processing. In healthy individuals, the N400 in response to homographs depends on the frequency of the homograph in the subjects’ L1 and L2 (Kerkhofs et al., 2006). Here, the authors used a semantic association paradigm in L2 with homographs as target words and observed a significant decrease in N400 amplitude in response to semantic priming but also in response to homograph frequency manipulation: the homographs with lower frequency in the subject’s L1 evoked an N400 with smaller amplitude compared to homographs with higher frequency. Furthermore, evidence suggests that the subject’s experimental environment (Elston-Güttler et al., 2005; Paulmann et al., 2006) and even his/her knowledge about the experimental manipulation (Khachatryan et al., 2016), can influence the N400 amplitude in response to the words related to inter-lingual homographs. For instance, in Elston-Güttler et al. (2005) and in Paulmann et al. (2006) the experimental environment was manipulated by showing a movie in the subject’s L1 or L2 before conducting the experiment in his/her L2. Elston-Güttler et al. (2005) showed that when the movie in L1 was shown prior to the experiment, at the beginning of the experiment, the target words that were L2 translations of the L1 meaning of the homograph (prime), evoked smaller N400s compared to the unrelated targets. Importantly, the homograph primes in this study were the last words of prime sentences such as, “The woman gave her friend a pretty GIFT” → POISON, where GIFT was the prime homograph meaning “poison” in German (subjects’ L1). This effect diminished and disappeared at the end of the experiment, which made the authors conclude that subjects needed to ‘zoom into’ their L2 in order to ignore the L1 meaning of the homograph. Paulmann et al. (2006) presented the same prime and target words outside the sentence context and observed the mentioned facilitation effect on the N400 across the experiment, concluding that if the context is not constraining enough, it is not possible to inhibit an individual’s L1. Unlike the previous studies, in Khachatryan et al. (2016) the experimental stimuli (word-pairs with homograph primes) were presented in subjects’ L1 only and, the effect of L2 interference in L1 processing was shown to depend on the experimental conditions. Here, it was shown that, when subjects suspected that their L2 knowledge is important for the experiment, the processing of words related to the L2 meaning of the homograph was facilitated. This was reflected by a decrease in N400 amplitude in response to those words. In all these studies, mainly the N400 ERP was evaluated. The P200 has never been studied in this context, except perhaps by Elston-Güttler et al. (2005), where the 150 – 250 ms time window (early time window) was investigated and a smaller negativity (N200) in response to targets related to the L1 meaning of the homograph was observed in the first part of experiment. They interpreted this observation as a facilitated processing of those words on the level of lexical access and orthographic processing.

In summary, to the best of our knowledge, we report for the first time on the interference of L2 in L1 processing in dominant bilingual (Dutch–English) patients with aphasia and in two control groups (young and age-matched with patients) using the ERP technique. The ERPs were recorded in response to four groups of word-pairs (244 word-pairs in total) following a cross-factorial design (relatedness × homograph use). Furthermore, we investigate their phonological/orthographic processing and lexical access in terms of the P200 (100 – 250 ms post-onset), and semantic processing in terms of the N400 (350 – 550 ms) in response to target words presented in the context of inter-lingual homographs (prime words). We hypothesize that, by using word pairs containing inter-lingual homographs as primes, we can evaluate the second (non-dominant) language interference in first (dominant) language processing in bilingual individuals with aphasia. Furthermore, by employing the ERP technique, we can find out which linguistic processes (i.e., lexical access and semantic processing) are affected by this interference. Following the literature, we expect to observe the effect of relatedness in healthy subjects during both the P200 and N400 time windows given that the priming can occur during both lexical access-reflected by P200 via automatic spreading activation (Collins and Loftus, 1975), and semantic processing-reflected by N400 via semantic priming (Van Vliet et al., 2014). As to the effect of homograph use and, consequently, the interference of L2 in L1 processing, we do not expect to observe the said interference in young healthy subjects, due to their good cognitive control that will most probably inhibit this interference. Most importantly, the subjects were informed in advance about the presence of words that could be related in their L2 and they were instructed to ignore them. In Khachatryan et al. (2016) it was shown that if subjects do not expect such a pattern in the stimulus set, but encounter it by accident (and realize the connection), the processing of such words is facilitated (decreased N400). The role of cognitive control in the processing of homographs and inhibition of one of the languages was previously suggested by Dijkstra et al. (1998), who tested homograph processing using behavioral responses only. In older healthy subjects this picture might change due to age-related decrease in cognitive control (Braver and Barch, 2002). In patients, we expect to observe a considerably more complex picture, since it was shown that language processing in bilingual individuals with aphasia depends on a complex interaction between a number of factors, such as, pre- and post-morbid language proficiency levels, age of language acquisition, level and location of impairment, etc. (Kiran and Lebel, 2007; Kiran and Iakupova, 2011). We predict that the pre- and post-morbid proficiency level of L2 will have a significant influence on the ERP pattern. We hypothesize that the higher the post-morbid L2 proficiency, the higher will be the L2 interference in L1 processing, given the impairment in executive control in those patients described in Verreyt et al. (2013). We predict that patients with higher pre-morbid L2 proficiency will have considerably more L2 interference in L1 processing during semantic processing (N400), since it has been shown that the information in patients with aphasia is not lost, but rather inhibited (Hagoort et al., 1996; Swaab et al., 1997, 1998).

If the proposed interference would exist, we expect the P200 and/or N400 (depending on pre- and/or post-morbid proficiency) in response to target words related to homograph primes to differ from the same ERPs in response to control target words. In both healthy individuals and patients with aphasia, this interference could be expressed in terms of a facilitation in word processing related to the L2 meaning of the homograph primes or an inhibition when the words are related to the L1 meaning of the homograph primes. However, in the opposite case, i.e., if there would be no interference of L2 in L1 processing, the ERPs in response to target words paired with the homograph primes should be similar to the ERPs in response to targets paired with control primes.

## Materials and Methods

### Subjects

Five post-stroke multilingual patients (four bilinguals and one trilingual) with aphasia (**Table 1**), five healthy bilingual (Dutch–English) students [average age 25.2 years old (std = 3.2), 3 females, one left-handed] from KU Leuven and seven older healthy bilingual (Dutch-English) individuals [average age 53 years old (std = 4.1), 2 females, all right-handed] age-matched to the patients participated in the study.

**Table 1:** Demographic and neurological characteristics of the patients with aphasia.

Patient	Gender	Age at the moment of evaluation (years)	Time since stroke (months)	Type of stroke (ischemia/hemorragia)	Neuroanatomic localization (MRI)	Aphasia Diagnosis	Rehabilitation period after stroke	Handedness (Left/Right)	Native Language
HB	M	65	41	Ischemia + hemorraghic transformation	Left temporoparietal	Amnestic	41	Right	Dutch
AC	M	73	20	Hemmoraghia	Left Temporal cortex	Amnestic	20	Right	Dutch
RL	M	49	33	Ischemia + hemorraghic transformation	Left frontal gyrus + caudate nucleus + insula	Amnestic	33	Right	Dutch
LVDS	F	49	18	Ischemia	Left insula + frontotemporal opercula + putamen + caudate nucleus	Wernicke	18	Right	Dutch
SG	M	58	30	Intracerebral bleeding	Left temporo-parietal, with impact on frontal cortex and thalamus	Wernicke	24	Right	German

Young healthy controls were KU Leuven students that were enrolled in a master program taught in English. Prior to their admission, these students had to provide a proof of English language proficiency. Age-matched controls were post-graduate workers or professors at KU Leuven who use English on a daily basis. As the current experiment consider word processing in a word-pair context, an advanced evaluation of our participants’ L2 knowledge was not deemed necessary. Instead, after the experiment, we showed our healthy subjects a list of the homographs used and asked them to indicate for which ones the English meaning was unknown. For the young healthy subjects the average number of unknown homographs was 3.2 (ranging from 1 to 5), for the older healthy subjects it was 3.7 (ranging from 2 to 7). Given that our healthy controls (both young and older) were teaching or taking classes in English (L2), we can confidently assume that their self-reported proficiency of English was as high as the ones of patients pre-morbidly.

Four out of five patients were Dutch-English bilinguals, while one patient (SG) was German–Dutch–English trilingual. All patients (except SG) had a mild impairment in their L1 (Dutch) and a moderate impairment in L2 (English). Patient SG, who had Dutch as L2 and English as L3 had a more severe impairment in both Dutch and English compared to the other patients. Their performance on the Aachen Aphasia Test (AAT) in Dutch and part 3 of the Dutch-English Bilingual Aphasia Test (BAT) for all patients except SG is shown in **Table 2**. Since for patient SG, Dutch and English were second and third languages, respectively, it was considered inappropriate to assess his linguistic abilities in these languages with the standard aphasia tests (e.g., AAT) designed to assess L1 linguistic abilities. Therefore, his knowledge of Dutch and English was evaluated using a subjective report on pre- and post-morbid proficiency (see further).

**Table 2:** The results of AAT presented in percentiles and 3rd part of the Dutch–English BAT for individual patients.

Test	Subtests	HB	LVDS	RL	AC
AAT^∗^	Token test	79	97	93	90
	Repetition	80	93	95	74
	Writing	97	100	100	99
	Naming	92	100	99	98
	Comprehension	98	98	100	96
BAT^∗∗^	Word recognition (10)	10	10	9	10
	Word translation (20)	9	11	17	5
	Sentence translation (36)	27	18	25	9
	Grammaticality judgment (16)	13	12	19	8

*^∗^Presented in percentiles, ^∗∗^presented the number of correctly responded items. The total number of items for each subtest of the BAT (maximum score) is presented between the brackets next to the subtest name.*

All patients were late dominant bilinguals that acquired English (L2) during their school years (around 12 years old) and further regularly used it in their work (on a daily basis) and more intensively when traveling. As said above, the results obtained with AAT might be misleading when used in L2, in particular for dominant bilinguals, which is the case with our patients. Instead, we employed a so-called language ability rating (LAR), which has been shown to be predictive for post-morbid performance in a number of linguistic tasks (Gray and Kiran, 2013; Kiran et al., 2014). The LARs for proficiency in English (L2 for 4 patients and L3 for patient SG) pre- and post-morbidly, as well as the frequency of pre- and post-morbid exposure to English are listed in **Table 3**.

**Table 3:** The subjective report on pre- and post-onset proficiency and frequency of English exposure.

	HB		LVDS		RL		AC		SG (L3)	
	Pre	Post	Pre	Post	Pre	Post	Pre	Post	Pre	Post
Reading proficiency	8	7	8	3	9	7	6	3	10	5
Writing proficiency	7	5	7	1	9	4	6	3	10	1
Speaking proficiency	7	4	8	3	9	5	6	2	10	4
Comprehension	7	5	7	2	9	7	7	6	10	6
Encountering frequency	9	5	9	3	4	4	4	3	8	2

*The scale ranges from 1 to 10 with 1 very poor and 10 perfect.*

The study was approved by the Ghent University Hospital’s and Leuven University Hospital’s ethical committees and was conducted in accordance with the latest version (2013) of the Declaration of Helsinki. Prior to participating in the experiment, the subjects were informed about its purpose, set-up and task, after which they were invited to sign the informed consent form.

### Materials

The stimuli consisted of four groups of word-pairs following a cross-factorial design (**Table 4**), crossing the factors of homograph use as prime and semantic/associative relatedness between prime and target words. All word-pairs were presented in Dutch only (L1 for four bilingual patients and L2 for patient SG). In total, 122 semantically and/or associatively related and 122 unrelated Dutch word pairs were used. The same prime word was paired once with a related target word and a second time with an unrelated one. In 61 word pairs from both related and unrelated word-pairs, the prime words were inter-lingual homographs.

**Table 4:** Definition of the stimulus groups FAS refers to the portion of subjects that answered with that particular word in response to the presented prime word.

		Use of homograph as prime	
		Homograph	Control
Semantic/associative relatedness	Related	Homograph-related Angel-bij (“sting - honeybee”) FAS_D_ = 0.13	Control-related Hond-poes (“dog - cat”) FAS_D_ = 0.13
	Unrelated	Homograph-unrelated^∗^ Angel-hemel (“sting-heaven”) FAS_D_ = 0.0004	Control-unrelated Hond-tafel (“dog-table”) FAS_D_ = 0

*^∗^FAS_E_ = 0.18. FAS_D_ refers to FAS in Dutch and FAS_E_ to FAS in English.*

The homograph-unrelated group (**Table 4**) consisted of unrelated word pairs in Dutch with the inter-lingual homograph as prime and the target chosen in such a way that its English translation was associated to the English meaning of the homograph (e.g., ‘angel → hemel’, meaning ‘heaven’ in Dutch). The homograph related group consisted of word pairs related in Dutch with the homograph as the prime word and the target associated to the Dutch meaning of the homograph (e.g., ‘angel’ → ‘bij’, in English ‘sting’ → ‘honeybee’). The other two groups were simple (control) Dutch word pairs with and without associations between prime and target words, such as ‘maand’ → ‘jaar’ (‘month’ → ‘year’) and ‘maand’ → ‘kust’ (‘month’ → ‘coast’). Finally, we adopted a balanced design with 61 word-pairs in each of the four presented groups.

The lexical characteristics of the target words did not differ significantly. Repeated measure ANOVA showed no significant difference between most of the studied stimulus characteristics of the four groups: word frequency [*F*(3,240) = 1.13, *p* = 0.34] was checked using the SUBTLEX-nl word frequency database (Keuleers et al., 2010), length [*F*(3,240) = 0.44, *p* = 0.73] and orthographic neighborhood size [*F*(3,240) = 0.63, *p* = 0.6] were checked using CLEARPOND non-commercial software (Marian et al., 2012). The only characteristic that showed a significant difference was concreteness [*F*(3,240) = 7.75, *p* < 0.0005]. It was checked using the concreteness rating database developed by Brysbaert et al. (2014). The concreteness values of target words on a 5-point scale (1 representing very abstract and 5 very concrete) in the homograph-related and homograph-unrelated groups were on average 3.6 and 3.7, while for the target words in the control-related and control-unrelated groups they were 4.2 and 4.3, accordingly. It is worth mentioning that the concreteness for targets between related and unrelated word-pairs with the same prime word did not differ significantly: for targets with homographs as primes, we had *F* < 1, *p* = 0.78 and for targets with control primes *F* < 1, *p* = 0.37.

The Forward Association Strength (FAS) of word pairs from the control-related (related group, Dutch control prime) and homograph-related (related group, homograph prime) groups were taken from the word association database of Dutch words developed by the Psychology Department of KU Leuven (De Deyne and Storms, 2008). Student’s t-test showed that the homograph-related and control-related groups did not differ in terms of FAS [*t*(1,120) << 1, *p* = 0.99]. The FAS values for the word pairs from the homograph unrelated group were taken from the Edinburgh Associative Thesaurus (EAT) (association strength database for English words) (Kiss et al., 1973). These values were extracted based on the English meaning of Dutch–English homographs (as primes) and English words (targets) that were most strongly related to the homographs. The homograph-unrelated stimulus group was compiled using the homograph words (e.g., ‘angel’) as primes and the Dutch translation of the English target words obtained from EAT (e.g., for prime ‘angel’ we added ‘hemel’ as target, which is the Dutch translation of ‘heaven’). Additionally, the English version of the homograph-unrelated group: the homographs as prime and the original English target words, were then used as related word-pairs in English (e.g., ‘angel’ → ‘heaven’) to evaluate the subject’s ability to conduct the experiment in L2 (English). Together with this group (English –related), a group of unrelated word-pairs in English with homographs as primes and English translations of randomly chosen words from the Dutch stimulus list as targets (e.g., ‘angel’ → ‘table’) was presented (see Experimental Design). The frequency of the English target words was checked using the American SUBTLEX database (Brysbaert and New, 2009), and it did not differ from that of the Dutch targets [*F*(1,364) < 1, *p* = 0.34].

### Experimental Design

Subjects were tested in a sound-attenuated, dimly lit room in the Laboratory of Neuro- and Psychophysiology (healthy controls) or the Ghent University Hospital (patients). Stimuli were presented on the LCD screen of a laptop (15′) viewed by the subjects at a distance of about 70 cm. Prior to the experiment, eye movements and eye blinks were recorded according to the aligned artifact average (AAA) set-up described in Croft and Barry (2000) and used to remove, in a later stage, the EEG artifacts caused by them (see further).

After that, subjects performed a semantic association judgment task. At the beginning of each trial, a cross appeared on the screen for a random duration between 500 and 700 ms, indicating that the subject should refrain from eye blinks or eye movements and fixate on the cross. Immediately after the cross disappeared, the prime word was shown for 500 ms. The target word was presented following the prime word for 500 ms with a jittered inter-stimulus interval within the range of 200–500 ms. Following the presentation of the target word, a blank screen appeared for 1000 ms followed by a screen with a question mark and two boxes, one labeled ‘Ja’ (‘yes’) and the other ‘Nee’ (‘no’). The subject was instructed to press the left button when he/she thinks there is an association between the words in Dutch or press the right button otherwise. This screen remained for 3 s or until the subject responded. Note that the button-press response was delayed, beyond the time window where the ERPs of interest (P200 and N400) are expected, to avoid contamination with response-related ERPs (Van Vliet et al., 2014). After pressing either button, subjects received feedback on their response: ‘geassocieerd’ (related) for the left button press and ‘niet geassocieerd’ (unrelated) for the right button press. The feedback did not reflect the correctness of the subject’s response but rather reminded them about the role of each button. For the healthy controls, the response hand was counter-balanced and patients performed the task with the hand unaffected by their neurological condition (post-stroke paresis). The explicit semantic association judgment task was chosen to increase the sensitivity of the N400 in response to both explicit and implicit semantic relatedness between words (in Dutch and English accordingly), as it was shown (Okita and Jibu, 1998; Kutas and Federmeier, 2009) that, although attention is not a necessary factor for N400 elicitation, it increases the sensitivity of the latter. In order to avoid possible confusion with words related in English (as the experiment was in Dutch), during the instructions we informed our subjects that some words might have a different meaning in English, but this should be ignored and attention should be paid to the Dutch meaning only. Hence, subjects were expected to judge word pairs accordingly and to respond to word associations in their L1.

All word pairs were presented in a pseudo-random manner. Each prime word was presented twice during the whole experiment and its presentation was counterbalanced so that half of the primes were first presented in the context of unrelated word pairs (stimuli from homograph unrelated and control unrelated groups), and the other half in the context of related word pairs (stimuli from homograph related and control related groups). We split the stimulus list into several short blocks and subjects could take a break every 5–7 min. Prior to the main experiment, each participant completed a short training session (six word pairs) on the same task, in order to familiarize the subject with the experimental paradigm and task.

At the end of the main experiment, participants performed another short block (English block) with the same semantic priming paradigm using stimuli from the homograph unrelated group translated into English and another set of unrelated English word pairs with the same homographs as prime words (see Materials, for a detailed description of the stimuli).

For patients only, in addition to semantic association judgment task with simultaneous EEG recording, paper and pencil tests consisting of AAT in Dutch and parts 1 and 3 of the Dutch-English BAT were implemented. The choice of parts 1 and 3 of BAT was motivated by the goal to investigate the interaction between two languages rather than the performance in each language separately, therefore, in order not to cause any further fatigue to our patients, we did not assess his/her knowledge of English separately. The order of the tests for the patients was counterbalanced, as for half of them the paper and pencil tests were performed first and for the other half EEG tests were performed first. Patients had approximately 30 min break between the two tests.

### Electroencephalogram Recording

EEG recording was performed using 32 active Ag/AgCl electrodes (BioSemi ActiveTwo) placed according to the international extended 10–20 system. Additionally, six external electrodes were placed, one on each mastoid, for further offline re-referencing, and four around the eyes, one on the upper and lower side of the left eye (vertical), and one near the external canthus of each eye (horizontal), for electro-oculogram (EOG) recording (bipolar recording). Except for the external ones, all electrodes were mounted in the electrode cap that was placed on the subject’s head. Conductive gel was applied in each of the electrode holes, as well as on the surface of the external electrodes, to reduce electrode impedance. The signal was recorded at a sampling rate of 2048 Hz. The impedance between the skin and electrodes was kept below 5 kOhm and the quality of the signal was constantly monitored.

The presentation of the experimental stimulus and the eye calibration session was performed using the non-commercial Psychophysics toolbox for Matlab (Brainard, 1997). The whole experiment with EEG recording, electrode cap mounting and explanation of the task took around 1 h for healthy subjects and approximately 2 h for patients given the additional paper and pencil tests and the 30 min break between them.

### Data Analysis

The EEG signal was re-referenced offline from the original common mode signal reference to an average mastoids reference and filtered using a 4th order Butterworth filter in the range of 0.5–15 Hz. Eye movement and blink artifact correction was performed with the AAA method described in Croft and Barry (2000) using the recorded EOG data. The EEG signal was segmented by defining epochs starting from 100 ms prior to the onset of the stimulus of interest (target word) until 1000 ms post-onset. In order to remove trials with remaining artifacts (residual eye movements, blinks and muscular artifacts), filtered epochs with an amplitude larger than ±50 μV at any recorded channel were discarded.

For the remaining trials, for each EEG channel, the baseline was subtracted using the average signal in the range starting from 100 ms prior to target onset till target onset (0 ms). Afterward, the areas under the curve in the range of 100–250 ms and 350–550 ms post-onset were calculated and taken as average amplitudes of the P200 and N400 ERPs, respectively.

### Statistical Analysis

For the behavioral data of English word-pairs, we conducted a repeated measure analysis of variance (ANOVA) with relatedness as independent factor and performance accuracy as dependent variable. For the Dutch part of the experiment, we conducted a separate ANOVA with the effects of relatedness (R), homograph use as prime (H) and their RxH interaction as independent factors, and performance accuracy as dependent variable.

As the Shapiro–Wilk normality test did not hold for our patient data, we applied the Kruskal–Wallis non-parametric test. Additionally, as the results on patient data depends (as it was mentioned earlier) on the number of individual factors (see section “Introduction”), we analyzed each patient separately.

We considered several factors in our analysis. First, we analyzed English word pairs separately to evaluate the effect of relatedness in both healthy controls and patients. Using Kruskal–Wallis test we compared amplitudes of each ERP (P200 and N400) in response to English related and unrelated word-pairs in order to observe a priming effect in our subjects in response to L2 stimuli.

For the Dutch stimuli, since non-parametric statistical tests cannot handle more than one factor when evaluating even slightly unbalanced data (slightly different number of trials per stimulus group obtained after cleaning the data), we evaluated each factor separately.

In order to assess the effect of interaction between homograph use as a prime and semantic/associative relatedness, i.e., as a substitute to the effect of interaction in a 2 × 2 designed parametric test (e.g., ANOVA), we first considered subgroups of word-pairs and evaluated the effect of homograph use in related and unrelated word-pairs independently. With this analysis, we studied the level and mechanism of L2 interference in L1 processing in healthy and patients with aphasia and, in this way, evaluated the possible presence of a facilitation of L2 meaning or an inhibition of L1 meaning of the homographs.

Afterward, we pooled the related and unrelated Dutch word-pairs and analyzed their ERP responses. Here, we evaluated the effects of homograph use (i.e., homograph prime versus control prime) and relatedness (i.e., related versus unrelated) independently. The research question for this analysis was whether in healthy controls or in patients, priming (relatedness effect) and use of homograph primes (effect of homograph use) can influence lexical access (P200 amplitude) and/or semantic processing (N400 amplitude) independently from each other.

For the Dutch and English stimuli taken together, we evaluated the effect of language (two levels, Dutch vs. English) on ERPs, and in this way assessed our subjects’ (in particular, our patients) abilities to discriminate between languages.

Given previous evidence (Lee et al., 2012) and the excellent temporal resolution of the ERP technique, we aimed to unravel which of the suggested factors influence lexical access (P200) and/or semantic processing (N400) during word-reading in a semantic association paradigm. Additionally, given our collected information on the patients’ bilingual abilities, we expected to be in a position to evaluate the role of those abilities in each of the mentioned processes. The results with *p*-values lower than 0.05 were considered statistically significant.

In order to detect significant correlations between ERP amplitudes of the early (P200) and late (N400) time windows and the patient’s subjective reports on pre- and post-morbid L2 proficiency and exposure frequency, we ran Spearman’s correlation between ERP amplitudes and scores of the subjective reports to account for non-normality of the data. We considered correlations with p-values below 0.05 statistically significant. Taking into account that we performed a correlation analysis on each individual electrode, the risk of observing false positive results on a single electrode is slightly higher; therefore, we report and discuss only the correlations that were significant on four or more electrodes.

## Results

### Behavioral Data

For young healthy subjects the average performance on all stimulus groups was 0.902, for age-matched healthy subjects 0.92 and for patients 0.76. For both groups of healthy subjects, as well as for patients, the lowest performance was on the related English word pairs (**Table 5**). When running repeated measure ANOVA on performance accuracy of English word-pairs with relatedness as independent effect, we observed a significant effect of relatedness for all three groups: young healthy *F*(1,8) = 8.69, *p* = 0.018, older healthy group *F*(1,12) = 17.14, *p* = 0.001, and patients (4 bilingual patients) *F*(1,6) = 56.5, *p* = 0.0003).

**Table 5:** Performance accuracy for each stimulus group for each patient separately and the two groups of healthy controls.

	Homograph unrelated (HUR)	Homograph related (HR)	Control related (CR)	Control unrelated (CUR)	English related (ER)	English unrelated (EUR)
LVDS	0.82	0.98	1	0.92	0.28	0.93
HB	0.82	0.87	0.97	0.95	0.57	0.95
AC	0.84	0.98	1	0.93	0.28	0.84
RL	0.79	0.88	0.97	0.98	0.41	0.95
SG	0.57	0.098	0.16	0.71	0.098	0.69
Age-matched control	0.87	0.95	0.96	0.98	0.81	0.94
Young control	0.86	0.94	0.96	0.94	0.74	0.97

A repeated measure ANOVA on performance accuracies for the Dutch stimulus list with homograph prime, relatedness, and their (RxH) interaction as independent factors showed a significant effect of homograph prime for young controls [*F*(1,16) = 16.18, *p* = 0.001], older controls [*F*(1,24) = 11.35, *p* = 0.0025] and patients [*F*(1,12) = 25.34, *p* = 0.0003]. The effect of relatedness was significant for the young healthy (*F* = 11.06, *p* = 0.0043) group and the patients (*F* = 16.31, *p* = 0.0016). The RxH interaction was significant only for the group of older healthy subjects (*F* = 9.72, *p* = 0.0047). The follow up Student’s t-test on behavioral results of age-matched controls with False Discovery Rate (FDR) correction for multiple comparisons (Benjamini and Hochberg, 1995) showed a statistically significant lower performance accuracy on homograph-unrelated group (**Table 5**) compared to other three groups (in all cases, *p* < 0.05). The performance accuracies on other stimulus groups did not differ statistically.

We did not include patient SG in the group analysis of performance accuracies as for him, Dutch was L2 and English L3. Therefore, it is reasonable that his performance for both languages was significantly lower compared to the other patients.

### Group Analysis of ERP Data

In what follows, we will report on statistically significant results on ERP data for both healthy subjects and patients.

**FIGURE 1:**
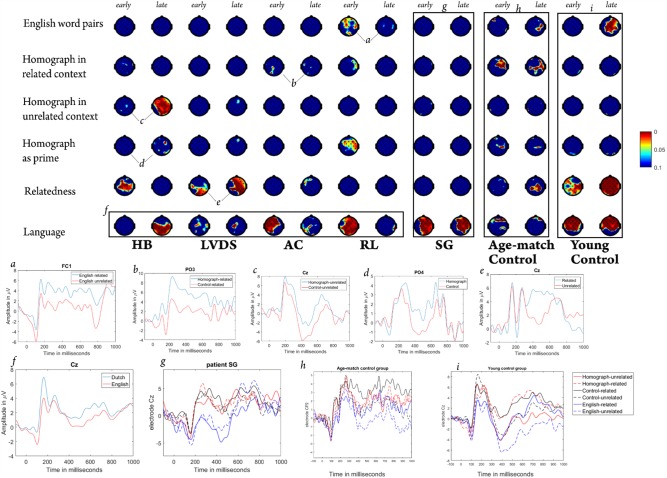
Scalp plots of word group effects (rows) for early (P200) and late (N400) ERP time-windows (columns) expressed in *p*-values. Panels *a* to *i* show ERP images of correspondingly labeled scalp plots. Columns labeled *early* and *late* are shown pairwise per patient and for the control group of healthy subjects. Shown are the scalp plots of the following effects: (1) English word-pairs = English-related vs. unrelated word-pairs; (2) Homograph in related context = difference between homograph-related and control-related word-pairs; (3) Homograph in unrelated context = the difference between homograph-unrelated and control-unrelated groups; (4) Homograph as prime = contrast between the average of homograph related and unrelated, versus the average of control-related and unrelated groups; (5) Relatedness = contrast between the average of homograph and control related versus the average of homograph and control unrelated word-pairs; (6) Language = difference between the average of all four Dutch word-pairs versus the average of two English word-pairs.

For the healthy young subject group (**Figure 1**, panel *i*), the English related word-pairs, evoked a significantly less negative N400 compared to unrelated word-pairs (for electrode Cz, *p* < 0.001). No significant difference between related and unrelated English word-pairs was detected in the early time window (P200). For Dutch word-pairs, the subgroup analysis did not reveal any significant effects of homograph use in any of the contexts (related or unrelated), for any time window. Thus, for this subject group, no significant difference was detected between homograph-related and control-related, and between homograph-unrelated and control-unrelated word-pairs. When related and unrelated Dutch word-pairs were pooled, the effect of homograph use (the comparison between words with a homograph and control primes) was more significant in the late time window. This effect was located posteriorly (for electrode O2, *p* = 0.016). Here, the effect of relatedness (comparison between the related and unrelated word-pairs independent of used prime) was significant for both time windows and was spread across the scalp (e.g., on electrode Pz, for early time window, *p* = 0.02, for late time window, *p* = 0.0001). For P200, the electrodes were: Fp1, AF3, F7, F3, FC1, FC5, T7, C3, CP1, CP5, P7, P3, Pz, PO3, O1, Oz, O2, PO4, P4, P8, CP6, CP2, C4, T8, FC6, FC2; and for N400 they were: Fp1, AF3, F7, F3, FC1, FC5, T7, CP1, C3, CP5, P7, P3, PO3, Oz, O2, PO4, CP6, CP2, C4, T8, P8, P4, FC6, FC2, F4, F8, AF4, Fz and Cz. When evaluating ERP responses to Dutch and English stimuli jointly, the effect of language (Dutch versus English) was significant for both time windows on a large number of electrodes (Fp1, AF3, F7, F3, FC1, FC5, T7, C3, CP1, CP5, P7, P3, Pz, PO3, O1, Oz, O2, PO4, P4, P8, CP6, CP2, C4, T8, FC6, FC2, F4, F8, AF4, Fp2, Fz, Cz for both time windows; e.g., on electrode Pz, for the early time window *p* < 0.0001, for the late time window *p* = 0.002).

As predicted, for the healthy age-matched subject group (**Figure 1**, panel *h*), the effects differed from the young healthy subjects and in general were more localized. Here, Kruskal–Wallis non-parametric test with relatedness as independent effect and ERP amplitudes (P200 and N400) in response to English target words showed a significantly smaller N400 amplitude in response to the targets in English related word pairs compared to the unrelated word pairs (for electrode CP2, *p* = 0.016). No difference between English word-pairs was found in the P200 time-window. For the Dutch stimulus, Kruskal–Wallis non-parametric test showed a significant effect of homograph use in the related context during both the early (AF3, F7, FC1, CP6, C4, FC6, FC2, F4, AF4 and Cz, e.g., on electrode FC1, *p* = 0.014) and late (AF4, F4, FC6, C4, CP2, CP6, P8, Pz, CP1, C3, AF3, e.g., on electrode PO4, *p* = 0.03) time windows. This effect was absent in the unrelated context, and when jointly evaluating the related and unrelated word-pairs of Dutch stimulus, the effect of homograph use as prime was present on electrodes Cz, C4 and AF3 during both the P200 (electrode C4, *p* = 0.027) and N400 (electrode C4, *p* = 0.045) time windows. Unlike the young healthy group, for the age-matched individuals, the effect of relatedness was mainly present in the late time window (electrode P4, *p* < 0.001) and was more localized on the right hemisphere (**Figure 1**, panel *h*). When analyzing Dutch and English stimuli together, we observed a significant effect of language during both the early (electrode CP2, *p* < 0.001) and late (electrode F8, *p* = 0.02) time windows, however, it was considerably more widespread in the early time window (**Figure 1**, panel *h*).

For the group of four patients who had Dutch as mother tongue (**Figure 1**, panel *f*), the effects of both language [χ^2^(1,1332) = 8.3, *p* = 0.004] and relatedness [for electrode Pz, χ^2^(1,909) = 7.83, *p* = 0.005] were significant in the P200 time-window. None of the other effects was significant. As a number of characteristics such as extent and nature of the impairment, as well as linguistic abilities can vary across patients, when lumping their data into a single group, valuable information might be lost. Therefore, we advocate evaluation of each patient separately, as done in the next section.

### ERP Data of Individual Patients

As we present each patient individually, to ensure that the ERPs evoked in response to stimuli are not confounded with noise and are considerably different from the baseline signal (general EEG “noise”), for each patient, we calculated a lower bound for the signal-to-noise ratio (SNR_LB_) of the whole epoch (starting from the stimulus onset till 1 s post-onset) expressed in dB compared to the baseline signal according to the approach described in Parks et al. (2016). The SNR_LB_ for each patient was as follows: HB – 7.4 dB, LVSD – 8.1 dB, AC – 6.1 dB, RL – 8.5 dB and SG – 5.4 dB. Since these values considerably exceed the threshold of 3 dB established by Parks et al. (2016) for the inclusion of a subject in the study, the signal quality of our patients’ EEG recordings can be considered as good.

To determine the patients’ ability to differentiate between items in their L2 (English), we evaluated the difference between English related and unrelated word-pairs. For English word pairs, the Kruskal–Wallis test showed a significant difference in the early time window between related and unrelated word-pairs for patient RL (for electrode Cz, χ^2^ = 3.92, *p* = 0.0477) with a larger positivity in response to related word-pairs (**Figure 1**, panel *a*). Patient HB, showed a significant effect of relatedness for English word pairs in the late time window, albeit for fewer electrodes (for electrode O1, *p* = 0.01), with a more negative N400 in response to unrelated word pairs.

To evaluate the L2 interference in L1 processing in different contexts for each patient individually, we implemented a subgroup analysis and verified the effect of homograph in related and unrelated contexts. The subgroup analysis with the Kruskal–Wallis test showed a significant effect of homograph use in related context (comparison between homograph-related and control-related groups) for 3 out of 4 patients (patient AC – for both the early and late time windows, *p* < 0.05, **Figure 1**, panel *b*; patients RL and HB – in both cases for the early time window only, *p* < 0.05). For the unrelated context, the effect of homograph use (comparison between homograph-unrelated and control-unrelated groups) was significant only for 1 out of 4 patients (HB) in the late time window (**Figure 1**, panel *c*) on multiple electrodes (all electrodes besides T7, CP5, P7, PO3, O1, O2 and F8; e.g., for electrode Cz, *p* < 0.01).

To evaluate the effect of relatedness between words in the patients’ mother tongue and the effect of homograph use independent of the context, we pooled the related and unrelated Dutch word-pairs and studied the effects of relatedness and homograph use as prime in the early (P200) and late (N400) time-windows. The effect of homograph use (comparison between the word-pairs containing homograph- and control- primes, independent from relatedness) was significant for 2 out of 4 patients with Dutch as L1: RL in the early time window (for electrode Cz, *p* = 0.03) and HB (**Figure 1**, panel *d*) in the late time window (for electrode PO4, *p* < 0.01). The effect of relatedness (comparison between related and unrelated word-pairs, independent from the used prime) was significant for 3 out of 4 patients: for patient HB in the early time window (for electrode Cz, *p* = 0.01), for patient AC in the late time window (for electrode FC5, *p* = 0.047) and for patient LVDS (**Figure 1**, panel *e*) in the both early (electrode Cz, *p* = 0.015) and late (electrode Cz, *p* = 0.002) time windows.

To study the patients’ ability to differentiate between L1 and L2, we combined Dutch and English stimuli and evaluated the effect of language (comparison between four Dutch and two English stimuli groups) using the Kruskal–Wallis test. This effect was significant for all patients (**Figure 1**, panel *f* for image of average ERPs across all patients), albeit to a different extent and in different time windows. For instance, for patient HB, this effect was more pronounced in the late time window on several electrodes distributed centroparietally (for electrode Pz, *p* = 0.003). For patient LVDS it was significant only on a few electrodes (CP1, C3, P3 and T8) in both time windows (in both cases, *p* < 0.05). For both patients AC and RL, this effect was more pronounced and widely spread across the scalp in the early time window (for electrode Cz, in both cases *p* < 0.005).

We additionally evaluated an ERP image (both for the early and late time-windows) of trilingual patient SG, who had Dutch as L2 and English as L3, using the same factors.

The ERP results (**Figure 1**, panel *g*) show that even though patient SG performed the task in both languages equally badly (**Table 5**), he could clearly differentiate between those two languages. This was deduced from the observed significant effect of language (P200 – χ^2^ = 12.68, *p* = 0.0004, N400 – χ^2^ = 15.44, *p* < 0.0001) for both time windows on a significant number of electrodes in the right hemisphere (**Figure 1**, panel *g*).

### Correlation Analysis

**FIGURE 2:**
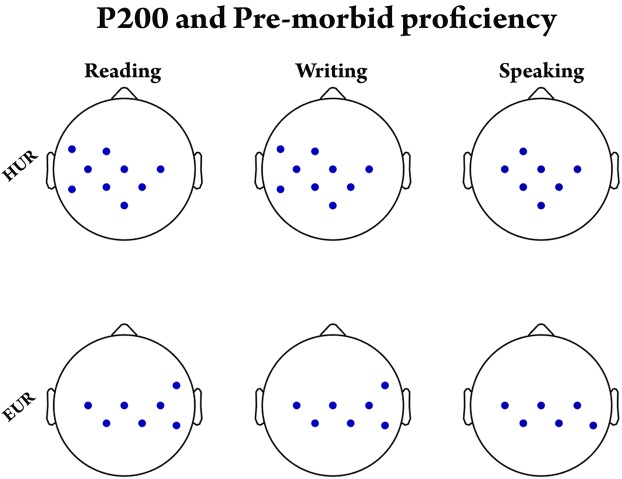
Significant correlations between amplitudes of P200 and subjective reports on pre-morbid L2 proficiency. Only the electrodes with significant correlations are shown. All patients are included in the correlation analysis. HUR, homograph-unrelated stimulus group; EUR, English-unrelated stimulus group.

Applying Spearman’s correlation between reports on pre- and post-morbid L2 proficiency and exposure, and ERP amplitudes, we revealed a number of significant negative correlations between P200 amplitudes in response to the homograph-unrelated and English-unrelated stimulus groups and a number of pre-morbid L2 characteristics (see **Figure 2**). None of the other correlations showed significance that met our inclusion criteria (i.e., four or more electrodes with *p* < 0.05). The correlation coefficients for significant values (*p* < 0.05) were below -0.95.

## Discussion

In the current study, we described, for the first time, the P200 and N400 ERPs in response to stimuli containing inter-lingual homographs in bilingual patients with aphasia. The only EEG study on bilingual patients with aphasia was the French-Farsi case presented by Radman et al. (2016), but they studied the effect of language training on the ERPs in response to L1 and L2 stimuli rather than the ERP picture of L2 interference in L1 processing.

Unlike behavioral studies that focus on one’s ability to process words as a whole, the EEG technique possesses the temporal resolution required to unveil language interaction as well as the processing of individual linguistic components (morphological and phonological processing, lexical access and semantic processing).

As indicator of L2 interference in L1 processing, we suggested the presence of significant effect of homograph use as prime in related or unrelated contexts. For our healthy young controls, as predicted, we did not observe this interference, as there was no effect of homograph in any of the contexts: no difference between homograph-related and control-related, or between homograph-unrelated and control-unrelated groups was present. We assume that the high level of cognitive control of young subjects is instrumental in inhibiting the alternative (L2) meaning of the homograph, which is unimportant to the task. This inhibitory effect of cognitive control on the processing of task-relevant and task-irrelevant stimuli was also previously suggested by Dijkstra et al. (1998) in their behavioral study. They showed no difference in reaction times in response to homographs and control words when performing a lexical decision task in one language only (L1). Thus, they concluded that the L2 meaning of the homograph was effectively inhibited due to cognitive control. We did observe a small posteriorly located effect of homograph in the late time window when jointly evaluating related and unrelated Dutch word-pairs, which we attribute to small differences in concreteness values of our targets that were paired with homograph and control primes. As expected, the relatedness effect in young subjects was present in both time windows, showing that the facilitation of word processing might already start during lexical access. This facilitation of word processing during lexical access can be explained by the activation of words (lexical level) related to the presented word as described in the automatic spreading activation theory (Collins and Loftus, 1975). This observation goes along with some previous monolingual studies that evaluated P200 modulation in response to a sentential stimulus (Dambacher et al., 2006; Lee et al., 2012) and regarded this ERP as an index of lexical access. They manipulated the position of the target word in the sentence, sentence predictability, and word frequency of the target word, and showed that the position of the word in the sentence and its frequency influenced the P200 amplitude, while sentence predictability mainly influenced the N400 amplitude. Summing up the results obtained from our young healthy subjects, we can conclude that they showed a durably effect of relatedness, reflecting the priming effect in one’s L1 and no effect of homograph in any of the contexts, therefore, no interference of L2 in L1 processing.

Unlike young healthy controls, the significant effect of homograph use in related context observed for the older healthy subjects indicates a considerable interference of L2 in L1 processing in this age group. Since we did not observe this effect in the unrelated context, we assume that the observed pattern is a result of an inhibition of the processing of L1 meaning of the homographs and the words related to that meaning, respectively. These results contradict some earlier studies that reported a facilitated processing of task-irrelevant meanings of homographs (Elston-Güttler et al., 2005; Paulmann et al., 2006). However, it is worth mentioning that these studies tested students, thus, they did not account for a possible decline in cognitive control in an older population (Barch et al., 2001; Braver and Barch, 2002). For these subjects, unlike for young controls, the effect of relatedness was mainly concentrated in the N400 time window, which indicates a postponement of facilitation of word processing until semantic processing (Kutas and Federmeier, 2011) in this age group.

As to the scalp distribution of the ERP components, for healthy young subjects, similar to the previous reports, we observed a priming effect spread over the scalp (Morris et al., 2010; Van Vliet et al., 2016). Specifically, for the P200 ERP (Fonaryova Key et al., 2005) a more frontal scalp distribution was suggested, while for the N400, a more lateralized one to the right (Kutas and Federmeier, 2011). Indeed, in our older healthy subjects, where the effects were spatially more constrained, the P200 ERP was observed more in frontal areas, while N400 in the centro-parietal areas, but with a bias toward the right hemisphere (**Figure 1**, panel *h*).

As to patients, we were expecting to obtain a complex picture due to the unique combination of their linguistic abilities and impairment characteristics. Indeed, we observed individual differences in their ERP patterns, something we could not observe from their behavioral data since they performed equally well on L1 and equally badly on L2. Patient SG, for whom none of the considered languages was the mother tongue, performed equally badly on both languages. However, his ERP data, similar to other patients, suggests SG’s ability to discriminate between those two languages (the effect of language was significant). In spite of being an extensive outlier, his data provides us with insight in the difference between mother tongue and second language beyond their morphological characteristics (Dutch as L1 for one patient, while L2 for the other one). Even though his pre-morbid proficiencies in both Dutch and English (L2 and L3) were comparable to his mother tongue, the brain lesion led to a significant impairment in these languages, while the ability to discriminate between them was spared. This observation confirms the previous suggestion (Ramus, 2002; Pierce et al., 2014) that the ability to discriminate between languages is rooted deeply in one’s brain. We suggest that it can be observed even if none of those languages is one’s mother tongue and the impairment is considerable.

Our results showed that the behavioral data of our patients was consistent across L1 stimulus groups, but the ERP image of each patient was unique. A number of studies (Kiran and Iakupova, 2011; Siyambalapitiya et al., 2013; Kiran et al., 2014) suggest that patient’s performance in L2 and L2 – L1 interference is an outcome of a complex interaction of impairment and pre-/post-morbid language proficiency. However, as they could not evaluate individual components of language processing, they were not able to come up with a more detailed explanation. In our case, due to the ERP technique, we were able to detect a pattern in their language processing (**Supplementary Figure S1**), which we will discuss further.

Statistical testing showed that, even when we combined all patient data, the observed effects of language (Dutch versus English) and relatedness in L1 in the early time window (100 – 250 ms, P200) was preserved, which is indicative of the robustness of these effects. The effect of language on both P200 and N400 was previously reported also for learners of L2 (Midgley et al., 2009). In our case, the English stimuli showed decrease in P200 amplitude even after the average across all patients with Dutch as L1 and English as L2. Since the P200 is shown to be affected by the individual’s alertness and selective attention, resulting in a decreased amplitude in case of increased attentiveness (Luck and Hillyard, 1995; Dambacher et al., 2006), it is logical to see a decrease in its amplitude for L2 compared to L1. This pattern was similar across patients and healthy individuals. The observed effect of relatedness on ERP was also previously reported in monolingual patients with aphasia with preserved comprehension (Hagoort et al., 1996). However, as the other effects disappeared after pooling patients, we advocated that we needed to investigate patients individually.

Kiran and Lebel (2007) observed a cross-linguistic semantic and translation priming effect only for some aphasia patients, but not for others. They, as well as Kiran and Iakupova (2011) suggested that this effect depended on the complex interaction between language proficiency, impairment and priming, which is normally a unique combination for each patient. Similarly, we obtained different results for different patients but unlike previous reports, here, by using EEG, we managed to evaluate the specificities behind lexical access and semantic processing of each patient and the influence of the considered factors on each of them. Compared to age-matched healthy bilinguals, in some patients we observed an effect of homograph use in unrelated context (difference between homograph-unrelated and control-unrelated groups), as well as when combining related and unrelated Dutch word-pairs on a larger number of electrodes during early (P200) or late (N400) time windows. These effects indicate facilitated processing of words related to the L2 meaning of the homographs. These specific patterns can be due to the unique combination of brain impairment and linguistic abilities (both pre- and post-morbid) of the individual patient. For instance, patients RL and AC had relatively little contact with their L2 (English) both pre- and post-morbidly (**Table 3**) and the influence of the considered factors was for those two patients mainly confined to the early time-window (P200) that reflects lexical access and the processing of phonological and orthographic information (Aerts et al., 2015). These are more basic components of language processing, and this early ERP was shown to be influenced by L2 already during relatively early stages of language acquisition (Shestakova et al., 2003; Liu et al., 2006). Thus, it is logical to observe a modulation of this ERP in patients with little pre- and post-morbid contact with L2. At the same time, reports on both pre- and post-morbid knowledge of English for RL and HB were significantly better than for the other patients. This was also confirmed by differences in ERPs in response to English stimuli (related versus unrelated) for those patients. Additionally, we observed a significant effect of homograph in these patients (see **Figure 1**), unlike the other patients, which indicates the presence of an interference of L2 in L1 processing, albeit in different time-windows. Since our subjects were explicitly instructed to ignore the possible English meaning of the presented stimuli, inhibitory control should play a significant role in the task performance and ERP patterns of those subjects. This was shown in the young healthy participants, and confirmed in the older healthy group, who exhibited, as predicted, a decrease in cognitive control (Braver and Barch, 2002; Grant et al., 2014) and therefore, a larger L2 interference. As the bilingual patients with differential aphasia (with one better improved/preserved language) experience impairment in cognitive control (Verreyt et al., 2013), the observed effect of the homograph use as prime in one of the contexts (related, unrelated or entire list) in almost all patients suggesting a significant interference of L2 in L1 processing is possibly a result of the mentioned control impairment. Importantly, unlike other patients, patient LVDS, who had a very low post-morbid L2 proficiency, performed the task mainly based on L1 (presenting the effect of relatedness) (**Figure 1**, panel *e*). This leads to the conclusion that her L2 was simply not strong enough to interfere with L1 processing.

Gray and Kiran (2013) showed that language awareness rating (LAR) is more representative of the performance of bilingual patients with aphasia than information about language acquisition (from LUQ – such as age of acquisition and so on). Here, we indeed showed that the subjective report on pre- and post-morbid linguistic proficiency in bilingual patients with aphasia can predict their ERP parameters, even when their behavioral responses are similar. Furthermore, we showed that the pre- and post-morbid L2 exposure frequency has a significant influence on the ERP pattern of individual patients. In healthy individuals, when evaluating the ERP in response to an artificial language with a gap in exposure (3 – 6 months), the syntax related ERPs (ELAN, LAN and P600) did not differ from the ones in response to the syntax of native language (Morgan-Short et al., 2012). However, the authors did not evaluate the ERPs in response to semantic violation, therefore, the role of language exposure in language processing and its effect on ERP pattern is still not well defined. Unlike patients RL and AC, patients HB and LVDS had a significantly larger exposure to English pre-morbidly, which dropped dramatically after the stroke incident (aphasia). In these two patients, we observed the effects mainly in the late time window (N400), which reflects the processing of semantic information (Kutas and Federmeier, 2011). The post-morbid L2 knowledge of HB was significantly higher compared to LVDS, who showed only a minor effect of language (difference between Dutch and English word-pairs) in both time windows. Therefore, a significantly facilitated processing of words related to the L2 meanings of inter-lingual homographs observed in patient HB was expected. However, it was observed only during semantic processing (N400). This was not the case with either group of healthy subjects. Hence, the facilitated processing on the semantic level can be the outcome of an even more pronounced impairment of cognitive control (inhibition) (Verreyt et al., 2013) and relatively spared language knowledge in those patients, which would lead to facilitated L2 processing in a condition when it is not required. Furthermore, similar to the temporal patterns of studied ERPs, their scalp distributions were specific to each patient – probably reflecting a unique combination of impairment and post-morbid compensation. This goes along with a previously reported shift (from right to left) in the N400 scalp distribution of monolingual patients with aphasia after an intensive speech therapy (Wilson et al., 2012). The observed shift was suggested to be the result of the compensatory mechanism behind speech recovery. Given that our patients also recovered their speech after the incident of aphasia, we can assume that their ERPs scalp distribution is partly due to the mentioned compensation.

Additionally, we observed a significant negative correlation between the subjective reports on pre-morbid L2 proficiency in a number of skills (reading, writing and speaking) and the P200 amplitude in response to homograph-unrelated and English-unrelated groups. This confirms that, as previously suggested (Gray and Kiran, 2013; Kiran et al., 2014), pre-morbid language proficiency rating can, to some degree, predict post-morbid performance in a number of tasks. It is worthy to note that these correlations were observed despite the relative consistency in task performance across patients, which is indicative of a superior sensitivity of ERPs compared to behavioral data. The correlations were present (**Figure 2**) between the rating of pre-morbid L2 proficiency and the stimulus groups that reflected L2 interference in L1 processing (homograph-unrelated) and partly L2 proficiency (English-unrelated). This result indicates interference of L2 in L1 processing on the level of lexical access in patients with higher pre-morbid L2 proficiencies, which is in accordance with the non-selective models of bilingual word recognition (BIA, BIA+, RHM) (van Heuven and Dijkstra, 2010). All these models suggest that when the word is presented in one of the languages it activates related words in both languages during different stages of word processing (i.e., orthographic, lexical access, semantic processing). Here, we confirm that the interference of L2 in L1 processing for bilingual patients with aphasia occurs during different stages of word processing, including lexical access and semantic processing.

## Conclusion

We investigated the interaction between two languages in bilingual patients with aphasia using inter-lingual homographs and electrophysiological activity (EEG-ERP) recorded when performing a semantic association judgment task in both languages. We showed that all our patients, even the one with Dutch and English as 2nd and 3rd languages (patient SG), could differentiate between these used languages. Unlike previous studies with behavioral data, by using the ERP method, we could observe significant effects of different factors in different time intervals (lexical access vs. semantic processing).

We showed that the strategies adopted for task performance and the processes underlying lexical access and semantic processing, gaged in terms of ERPs, depend not only on pre- and post-morbid proficiency levels (**Supplementary Figure S1**), but also on the frequency of pre- and post-morbid L2 exposure.

## Author Contributions

EK and MVH designed the study. MDL and KDK recruited patients and conducted paper and pencil aphasia tests on patients and analysis of data from aphasia tests. EK conducted EEG study and EEG data analysis, as well as statistical analysis of the whole data. BW recruited and conducted EEG study on age-matched healthy individuals. EK and MVH wrote the manuscript. All co-authors revised manuscript and had their contribution in creating the final version of the manuscript.

## Conflict of Interest Statement

The authors declare that the research was conducted in the absence of any commercial or financial relationships that could be construed as a potential conflict of interest.

## Supplementary Material

FIGURE S1 Schematic representation of second language (L2) interference in first language (L1) processing in bilingual patients with aphasia and factors influencing this interference.
