# Supplementary figures and images for: Event Related Potential Study of Language Interaction in Bilingual Aphasia Patients
Source: Front Hum Neurosci. 2018 Mar 5;12:81. doi: 10.3389/fnhum.2018.00081 (PMC5844919; doi:10.3389/fnhum.2018.00081)

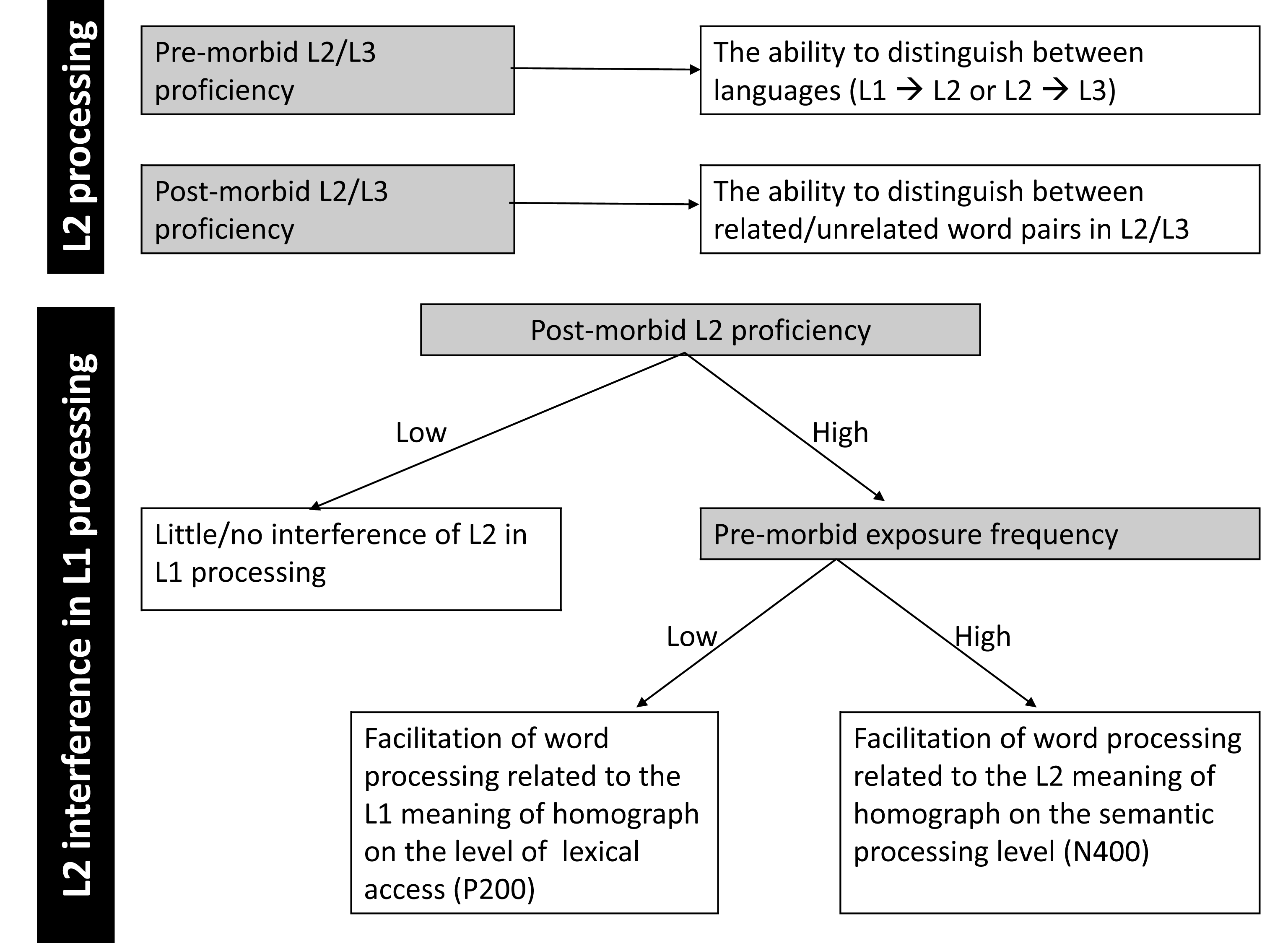

Supplement: FIGURE S1 — Schematic representation of second language (L2) interference in first language (L1) processing in bilingual patients with aphasia and factors influencing this interference. [file Image_1.TIF]
